# Integrated transcriptomic and metabolic profiling reveals the molecular mechanism of improved nitrogen metabolism in walnut (*Juglans regia* L.) roots mediated by soybean intercropping

**DOI:** 10.3389/fpls.2025.1658364

**Published:** 2026-01-29

**Authors:** Jing Ren, Xiaoyong Liu, Yifeng Wang, Xuefeng Jiang, Yaonian Chen

**Affiliations:** 1 Institute of Forestry, Fruit and Flower Research, Gansu Academy of Agricultural Sciences, Lanzhou, China; 2 Technique College of Agriculture and Forestry, Longnan Normal University, Longnan, Gansu, China

**Keywords:** walnut and soybean intercropping, nitrogen metabolism, transcriptome analysis, metabolomics analysis, multi-omics

## Abstract

**Introduction:**

The practice of intercropping young walnut orchards with soybeans has emerged as a promising approach within the framework of sustainable agricultural management. However, the precise impacts of soybean intercropping on nitrogen metabolism in walnut roots remain insufficiently elucidated.

**Methods:**

In this study, ‘Yuanlin’ walnut and ‘Longhuang 3’ soybean were used as experimental materials, with two planting patterns established: walnut monoculture and walnut-soybean intercropping. A combination of metabolomics, transcriptomics (RNA-seq), and physiological index determination was employed to systematically investigate the regulatory mechanisms of nitrogen metabolism in walnuts under the intercropping system.

**Results and discussion:**

The results showed that the dry matter accumulation of both aboveground tissues and roots in intercropped walnuts was significantly higher than that in monocropped walnuts, with the root system had a distinct vertical growth advantage. The nitrogen content in aboveground portions during dormancy and in roots during the hard kernel stage and dormancy was markedly elevated compared to monoculture systems. Metabolomics analysis revealed that differential metabolites in walnut under intercropping are significantly enriched in the carbon-nitrogen metabolic pathways and nitrogen transmembrane transport pathways. RNA-seq analysis identified 3,978 differentially expressed genes (DEGs), with significantly enrichment in the “nitrogen utilization” pathway. Furthermore, integrated analysis indicated that nitrogen metabolites may play a significant role in the walnut intercropping system. Key genes associated with nitrogen metabolism (*NR*, *NIR*, *GOGAT*, *GDH*, *NRT*, and *AMT*) exhibited significant alterations under the intercropping system. Enzyme activity validation demonstrated that intercropping substantially enhanced the activities of GS, GOGAT, and other enzymes, thereby strengthening the GS/GOGAT cycle responsible for converting inorganic nitrogen into organic nitrogen. This study confirms that walnut-soybean intercropping promotes dry matter accumulation and nitrogen allocation by activating root carbon-nitrogen metabolic pathways and the expression of nitrogen metabolism-related genes. These findings provide critical metabolic and transcriptional evidence supporting the sustainable development of intercropping systems in dryland orchards.

## 1 Introduction

Nitrogen is an essential mineral nutrient for plant growth, serving as a key component in nucleic acids, proteins, and hormones (Anas et al., 2020). Plant nitrogen metabolism constitutes an integrated network. In leaf cells, nitrate is first converted to nitrite by nitrate reductase (NR), and then reduced to ammonium nitrogen by chloroplastic nitrite reductase (NIR) and cytosolic glutamine synthetase (GS) (Ashraf et al., 2018; Tegeder and Masclaux-Daubresse, 2018). Ammonium nitrogen originates from nitrate reduction and root uptake of exogenous nitrogen via ammonium transporters (AMT). It is assimilated into organic compounds through the action of glutamate synthetase (GOGAT), which combines it with α-ketoglutarate from the tricarboxylic acid cycle. The GS/GOGAT cycle converts α-ketoglutarate and ammonia into glutamate (Temple et al., 1998). Glutamate dehydrogenase (GDH), a key enzyme in carbon-nitrogen metabolism, catalyzes the reversible conversion of α-ketoglutarate and ammonia into glutamate to maintain metabolic balance (Prakash et al., 2018). This metabolic process underpins crop nitrogen uptake and utilization, and directly impacts agricultural nitrogen cycling and yield formation (Masclaux-Daubresse et al., 2010). However, the excessive use of chemical nitrogen fertilizers in modern agriculture has led to increased costs, reduced nitrogen use efficiency, and environmental issues, including greenhouse gas emissions, soil acidification, and ecosystem imbalance (Kim et al., 2022; Govindasamy et al., 2023). Therefore, optimizing nitrogen management strategies to enhance efficiency and mitigate pollution is crucial for fostering sustainable agriculture.

In addition to the application of nitrogen fertilizers, planting patterns also play a critical role in influencing nitrogen use efficiency and crop yields (Azam et al., 2024). Intercropping, a planting practice involving the simultaneous cultivation of two or more crops in the same field during the same growing season, can enhance the utilization efficiency of light, temperature, water, atmospheric resources, and nutrient resources through interspecific ecological niche complementarity among species. This significantly improves resource utilization efficiency and land productivity (Nasar et al., 2024). For instance, intercropping systems such as maize/soybean (Raza et al., 2022), Chinese chestnut/tea (Wu et al., 2021), and cereal/legume (Demie et al., 2022) in China have demonstrated increased crop yields and quality by optimizing resource allocation. From a soil ecological perspective, intercropping systems can enhance nitrogen supply capacity and promote plant absorption by accelerating the soil mineralization-immobilization turnover process, thereby increasing food production (Xu et al., 2018). Studies have shown that walnut intercropping systems maintain higher levels of soil organic carbon and total nitrogen (TN) compared to monoculture systems (Lu et al., 2015). Furthermore, walnut-tea intercropping improves the adaptability of host plants by regulating the soil microbial community structure (Bai et al., 2022). When selecting intercropping plant materials, legumes play a pivotal role due to their ability to fix biological nitrogen. This not only provides additional nitrogen resources to the ecosystem but also supplies nitrogen to non-fixing plants through residue decomposition and root exudates (Mayer et al., 2003; Mcneill and Fillery, 2008). Especially in the context of rising costs of chemical nitrogen fertilizers and increasing environmental pollution, the importance of legumes in future tree intercropping systems will become even more pronounced (Vejan et al., 2021). However, systematic research on the mechanisms by which walnut intercropping systems affect biological nitrogen fixation and nitrogen metabolism in legumes remains insufficient.

Walnut (*Juglans regia* L.), also referred to as Persian walnut, is among the “four major nuts globally” (Abdallah et al., 2015). As the world’s largest walnut producer, China extensively employs intercropping system that integrate walnuts with leguminous plants in dryland agricultural regions. Although soybean intercropping is a dominant practice in young walnut orchards in Gansu, the influence of leguminous nitrogen fixation on walnut root systems and the underlying molecular regulatory mechanisms remain underexplored. This study employs Liquid Chromatography-Mass Spectrometry (LC-MS) technology to systematically compare the metabolic profiles of walnut roots under monoculture versus intercropping conditions. Additionally, transcriptomic analysis is utilized to investigate the expression patterns of key genes associated with nitrogen metabolism across different planting systems. These findings are intended to provide a solid scientific basis for elucidating the nitrogen metabolic mechanism in the walnut-soybean intercropping system.

## 2 Materials and methods

### 2.1 Plant materials and experimental

**Figure 1:**
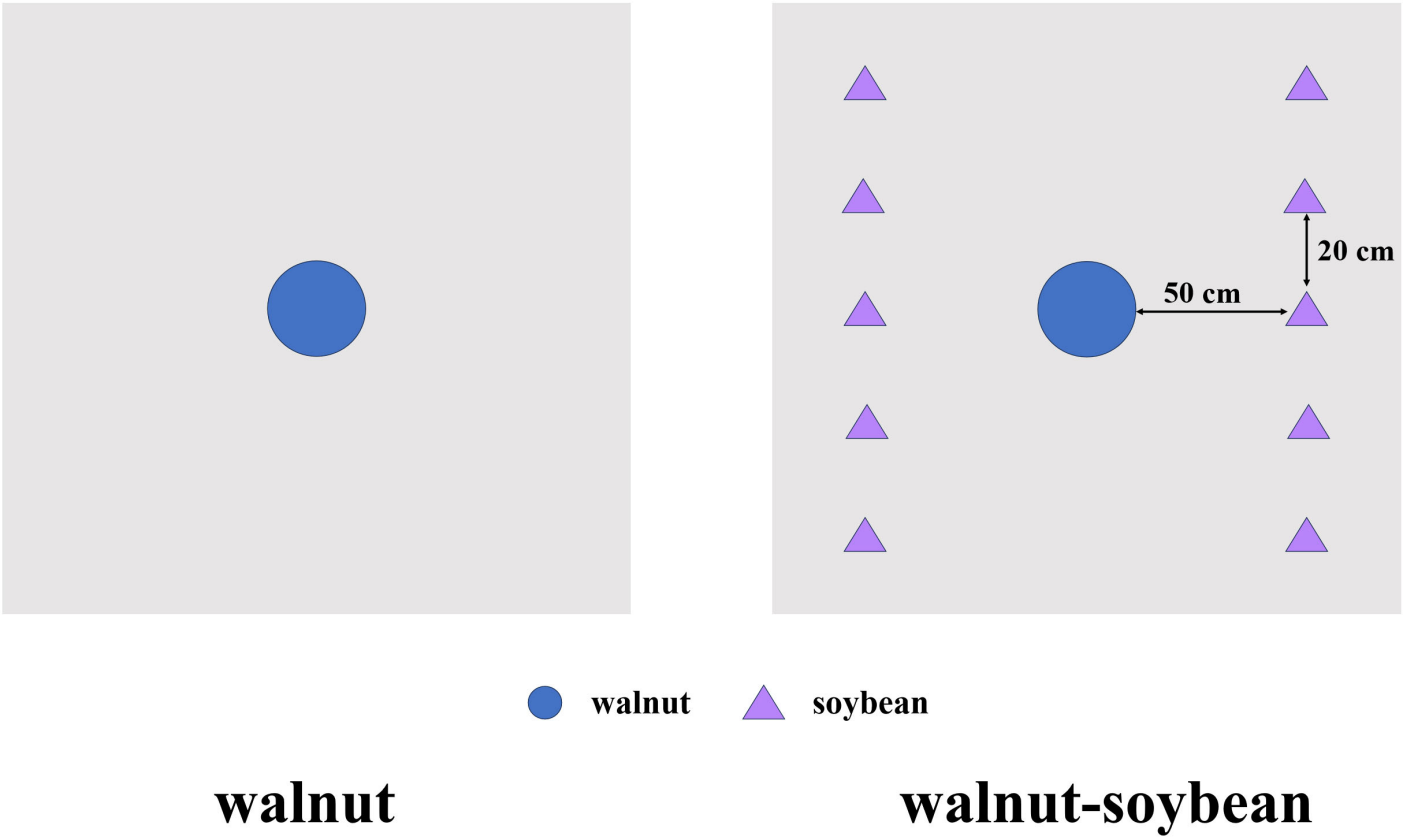
The cropping patterns of walnut monocropping and walnut*-*soybean intercropping.

The experiment was conducted at the main fruit tree germplasm resource garden of the Gansu Academy of Agricultural Sciences (N 36°6’, W 103°41’, altitude 1530 m). This region is characterized by an average annual rainfall of 329 mm, an average annual temperature of 9.6°C, an extreme minimum temperature of -25°C, and a frost-free period of 196 days. The experimental soil was classified as loessial, with the preceding vegetation consisted of three-year-old dandelions in a fallow orchard plot. Soil analysis of the topsoil layer (0-20 cm) revealed the following properties: organic matter content, 22.3 g/kg; total nitrogen (TN), 1.45 g/kg; total phosphorus (TP), 1.18 g/kg; total potassium (TK), 22.06 g/kg; available nitrogen (AN), 117.71 mg/kg; available phosphorus (AP), 28.90 mg/kg; available potassium (AK), 334.30 mg/kg; and pH, 8.37. Prior to the experiment, topsoil samples were collected and sieved to remove debris for subsequent analysis. One-year-old grafted walnut seedlings of the “Yuanlin” cultivar—grafted onto rootstocks derived from native late-fruiting thick-shelled walnuts in Cheng County, Longnan City, Gansu Province—were transplanted on March 25, 2019. The soybean cultivar “Longhuang 3”, a locally dominant variety, which was sown on April 15, 2020. Both cultivars are widely cultivated in the study region. Two planting systems were established: walnut monoculture and walnut-soybean intercropping (**Figure 1**). The experiment was conducted using planting bags (140 cm × 140 cm, 1.96 m²) as individual experimental units (plots). The walnut trees had a main trunk height of 60 cm and a crown width of 80 cm. In the walnut-soybean intercropping system, soybeans were sown in a single north-south row on both the east and west sides, with a row-to-trunk distance of 50 cm. Intra-row spacing of soybean plants was 20 cm, resulting in 10 plants per planting bag. A completely randomized design was adopted, with nine biological replicates per treatment. All experimental units were subjected to local conventional agricultural management practices (e.g., irrigation, weeding) without additional nitrogen fertilization.

### 2.2 Sampling and index measurement

Sampling was conducted at three critical stages: the hard kernel stage of fruit development (June 25, 2020), the oil synthesis period (August 15), and before walnut leaf abscission (October 5). Destructive sampling of whole plant was performed, with walnuts divided into four components (main trunk, branches, leaves, and root system) and soybeans divided into three components (pods, straw, and root system). After transporting the samples to the laboratory, the fresh weight of each component was measured. Subsequently, the samples were blanched at 105°C for 30 minutes and then dried at 80°C until a constant weight was achieved. Following cooling, the dry weight of each component was recorded. A portion of the dried samples was ground, passed through a 0.25 mm sieve, and homogenized for storage. The total nitrogen content of the plant samples was determined using the Kjeldahl method. Soil samples collected from the walnut rhizosphere were promptly transported to the laboratory for air-drying, grinding, and sieving before subsequent analyses. The TN, total phosphorus (TP), and total potassium (TK) contents of the soil were determined using the automatic nitrogen determination method (Li et al., 2016), the molybdenum-antimony colorimetric method (Wang et al., 2014), and the sodium hydroxide fusion method (Liu et al, 2023), respectively. Soil organic matter (SOM) content was determined via the potassium dichromate oxidation method (Liu et al., 2023). Soil available nitrogen (AN), available phosphorus (AP), and available potassium (AK) were determined using the alkali diffusion method (Razgulin, 2009), the sodium bicarbonate extraction followed by molybdenum-antimony-ascorbic acid colorimetric method (Mussa et al., 2009), and the neutral ammonium acetate extraction method (Wang et al., 2014), respectively.

### 2.3 Metabolomic analysis

The metabolite extraction was conducted using the organic reagent-based protein precipitation method, and quality control (QC) samples were prepared simultaneously. The QC samples were generated by pooling equal volumes of the prepared experimental samples. The extracted samples were randomized in sequence, with QC samples interspersed before, during, and after the sample set to evaluate the reproducibility of the experimental technique (Dunn et al., 2011). Mass spectrometry analysis was performed in both positive and negative ion modes. Raw data acquired from the TripleTOF5600plus mass spectrometer (SCIEX, Framingham, MA) were converted into a readable mzXML format using the MSConvert software from ProteoWizard. Peak detection and alignment were carried out using XCMS software, followed by quality control of the peak extraction process. Adduct ions of the detected compounds were annotated using CAMERA, and preliminary identification was conducted using the metaX software (Wen et al., 2017). During the identification process, both primary and secondary mass spectrometry data were matched against a self-constructed standard compound database. Candidate metabolites were further annotated using public databases such as Human Metabolome Database (HMDB 5.0: https://hmdb.ca/) and Kyoto Encyclopedia of Genes and Genomes (KEGG: https://www.kegg.jp) to elucidate their physicochemical properties and biological functions. Differential metabolites were quantified and screened using the metaX software.

### 2.4 Transcriptome analysis

In this study, six samples were sequenced on the Illumina NovaSeq 6000 sequencing platform (Illumina, San Diego, CA), and the raw sequencing data were analyzed by Shenzhen Huada Gene Technology Service Co., Ltd. Initially, total RNA was extracted from the samples using a plant RNA extraction kit (R0017M, Beyotime), and its concentration, purity, and integrity were evaluated using a NanoDrop spectrophotometer, Qubit^®^ 2.0 fluorometer, and an Agilent 5400 bioanalyzer, respectively. Subsequently, mRNA was enriched from total RNA, fragmented, and reverse-transcribed into cDNA using random hexamer (N_6_) primers. After second strand cDNA synthesis, the double-stranded DNA was subjected to end repairs, phosphorylation, and 3’-A overhang addition, followed by ligation with bubble adapters. The ligation products were amplified by PCR, denatured to single-stranded DNA, and circularized to construct libraries for DNBSEQ sequencing.

The raw data generated from sequencing are referred to as raw reads. To ensure data quality, low-quality reads, adapter contamination, and reads with excessive unknown base ‘N’ content were filtered out, resulting in clean reads. The clean reads were aligned to the reference genome for new transcript prediction, SNP and InDel detection, and alternative splicing gene analysis. Newly identified transcripts with protein-coding potential were integrated into the reference gene sequences to form a comprehensive reference sequence set. Gene expression levels were subsequently calculated. Differentially expressed genes (DEGs) were filtered using thresholds of |log2 (fold change)| ≥ 1 and false discovery rate (FDR) < 0.05. Finally, DEGs between different samples were detected as required, and in-depth clustering and functional enrichment analyses were conducted. Pearson correlation coefficients were used to assess the biological variability of DEG expression among samples, where an R^2^ value closer to 1 indicates a stronger correlation between samples.

### 2.5 Quantitative real-time PCR

To validate the reliability of the root transcriptome sequencing results, qRT-PCR was performed on six genes associated with nitrogen metabolism in the roots. First-strand cDNA synthesis was carried out using the HiScript II 1st Strand cDNA Synthesis Kit (R212-01, Vazyme Biotech). Subsequently, qRT-PCR was executed on the Bio-Rad CFX96^™^ Real-Time PCR Detection System (Bio-Rad), employing ChamQ SYBR Color qPCR Master Mix (Q411-02, Vazyme Biotech). The relative expression levels of the target genes were calculated using the 2^-ΔΔCt^ method. The primers used for qRT-PCR analysis are listed in **Supplementary Table S1**.

### 2.6 Statistical analysis

All experimental data were statistically analyzed and subjected to one-way analysis of variance (ANOVA) using SPSS 22.0 software. For multiple comparisons following ANOVA, Tukey’s honestly significant difference (HSD) test was used to determine statistical significance (*P* < 0.05). Graphs were generated using GraphPad Prism 8.3 software.

## 3 Results

### 3.1 The differences in dry matter accumulation between different planting patterns

**Figure 2:**
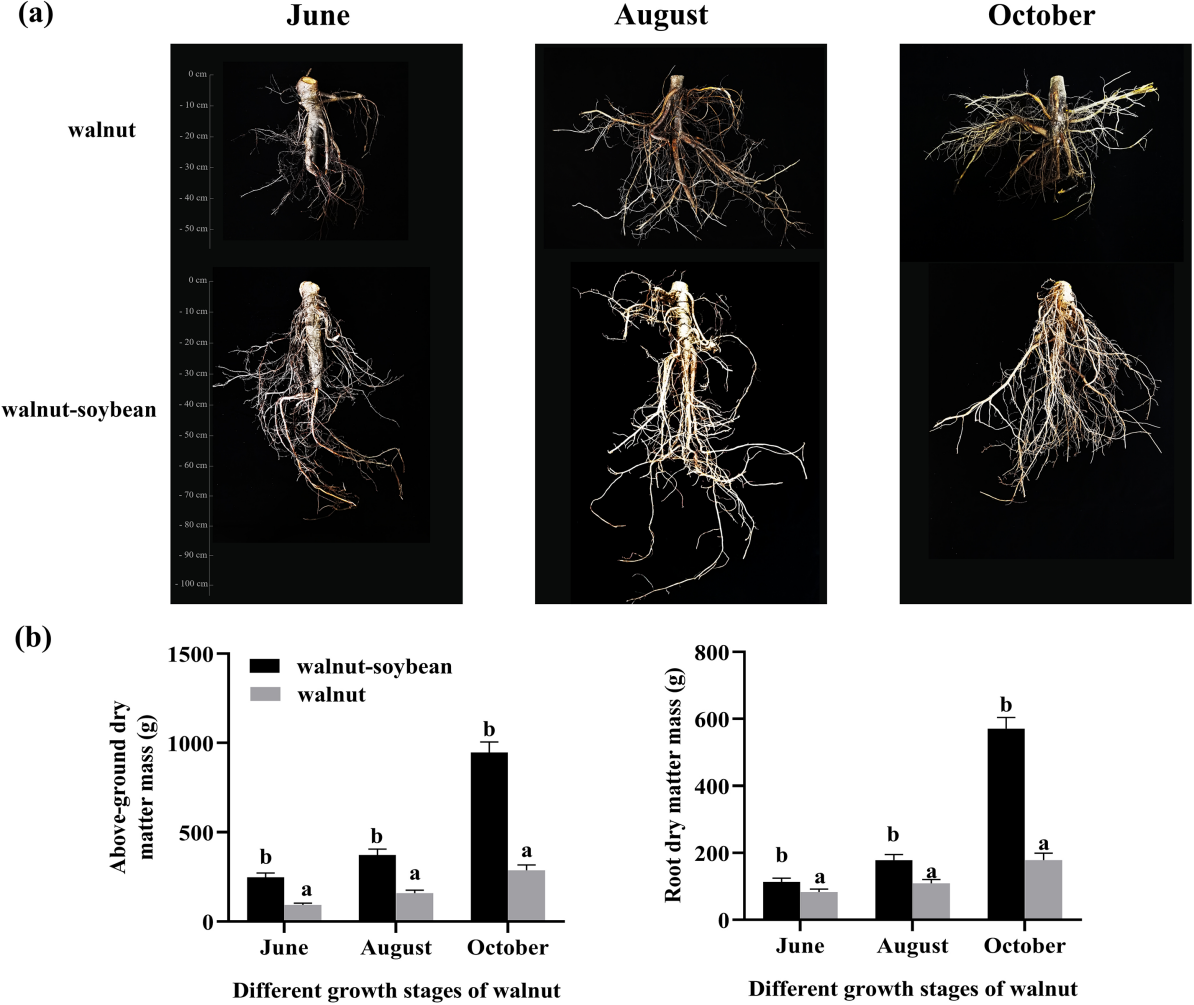
The influence of intercropping on the dry matter accumulation of walnut. **(A)** Effects of intercropping on the root morphology of walnut during the hard kernel period (late June), oil synthesis period (mid-August), and before leaf abscission (early October). Walnut: monocropped walnut roots; Walnut-soybean: intercropped walnut roots. **(B)** Dry matter accumulation of aboveground and root parts of walnut under different planting patterns. The lowercase letter represent significant differences at the same growth stage (*p* < 0.05).

From the observation of root system morphology (**Figure 2A**), significant differences in walnut root architecture were identified under different planting patterns. Specifically, the vertical roots of intercropped walnut progressively extended downward during growth and exhibited more pronounced development compared to horizontal roots. In contrast, the root distribution of monocropped walnut was relatively uniform. To accurately quantify these morphological differences, further determinations of aboveground and root dry matter accumulation were conducted.

Under both planting patterns, the dry matter mass of both aboveground and root components consistently increased, peaking in October. Notably, starting from August, the rate of dry matter accumulation accelerated significantly (**Figure 2B**). Compared with June, by October, the aboveground dry matter accumulation of intercropped and monocropped walnuts increased by 382.16% and 309.11%, respectively, while the root dry matter mass increased by 505.41% and 215.75%, respectively. It is worth noting that the total dry matter accumulation of intercropped walnut was higher than that of monoculture walnuts, indicating that intercropping has a significant advantage in promoting dry matter accumulation.

### 3.2 Nitrogen allocation and soil nutrient under different planting patterns

**Figure 3:**
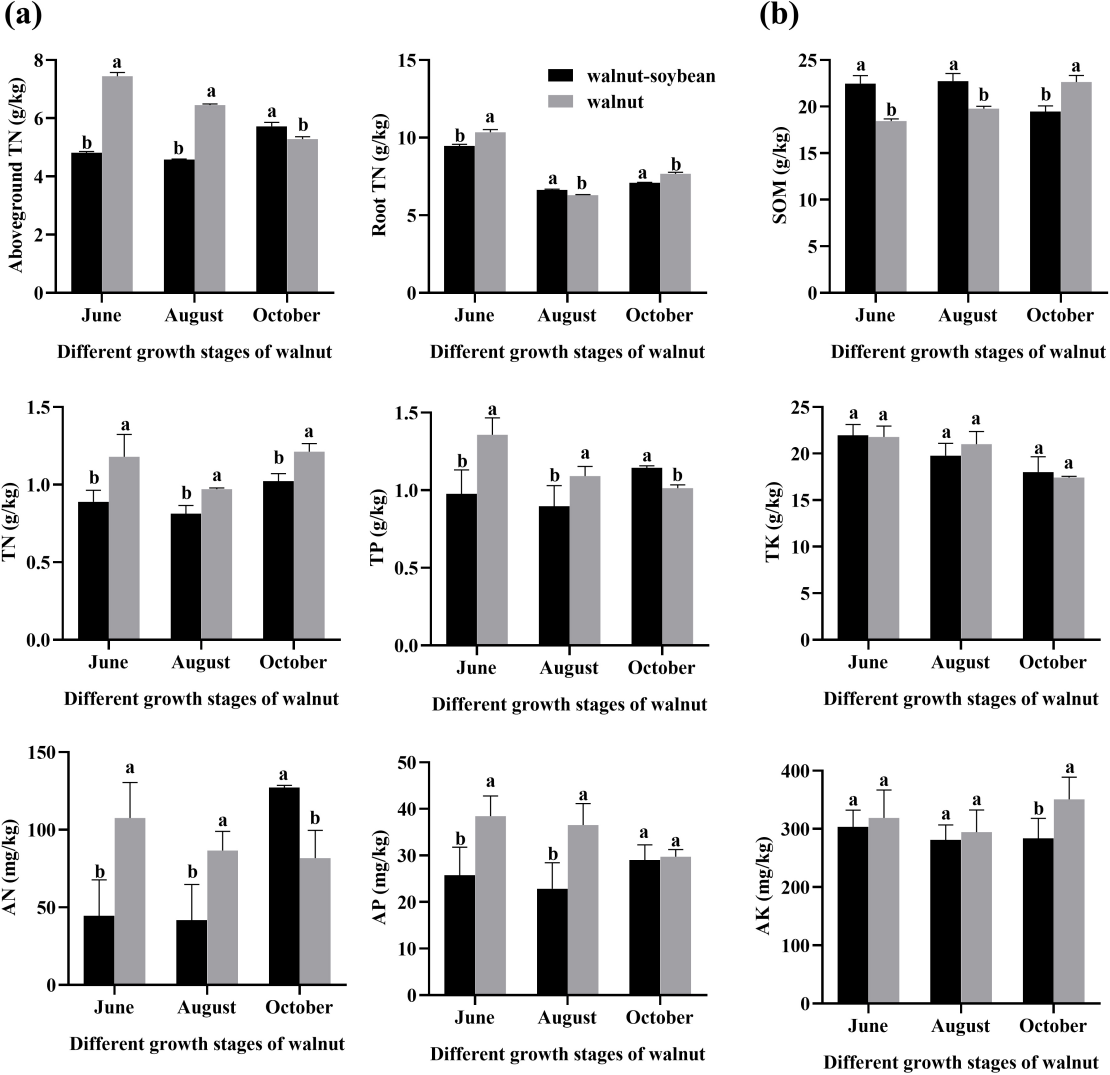
Nitrogen content of walnut roots and soil nutrient contents under different planting patterns. Walnut-soybean denotes the intercropping pattern, and walnut denotes the monoculture system of walnuts. **(A)** Nitrogen content of aboveground parts and roots. **(B)** Organic matter, total nitrogen, total phosphorus, total potassium, available nitrogen, available phosphorus, and available potassium contents in soil are respectively shown. Different lowercase letters indicate significant differences at the same growth stage (*p* < 0.05).

In cereal-legume intercropping systems, leguminous crops can reduce reliance on chemical nitrogen fertilizers through biological nitrogen fixation and transfer a portion of nitrogen to neighboring cereal crops. This provides an additional nitrogen source for the growth and development of cereal crops (Kebede, 2021; Landschoot et al., 2024). However, the nitrogen allocation in walnut root within walnut-soybean intercropping systems remains poorly understood and requires further investigation. Our results showed that during the hard kernel period and nut maturity period, the nitrogen content in the aboveground component of intercropped walnuts with soybeans was lower than that in monoculture. This phenomenon may be related to potential interspecific interactions: the nitrogen-fixing capacity of soybeans (a key characteristic in such intercropping systems) and the likely prioritization of nutrient allocation to developing walnut fruits could collectively contribute to the observed difference, though this requires further verification through targeted nutrient allocation studies. Conversely, during the dormant period, the nitrogen content in the aboveground parts of walnuts in intercropping is higher than in monoculture, which may be related to the nutrient retranslocation to the tree body and the continuous absorption by the root system. Similarly, the nitrogen content in the roots of walnut trees exhibits a comparable trend, being lower than in monoculture during the nut maturity period but higher during the hard kernel period and dormant period (**Figure 3A**).

Intercropping leguminous plants in orchards increases soil organic carbon, microbial carbon, and total nitrogen contents. Compared with monoculture, intercropping increased SOM content during the hard kernel stage and nut maturity stage but decreases it during the leaf-falling stage, ensuring a continuous nutrient supply for walnut growth (**Figure 3B**). During the hard kernel stage and nut maturity stage, intercropping reduces TN, TP, AN, AP, and AK contents in the soil compared to monoculture. This observation reflects intensified soil nutrient utilization in the intercropping system. During the dormant period, as walnut nutrient absorption decreased, soil nutrients are able to recover. Furthermore, after soybean harvest, soil nutrient contents in both intercropping and monoculture systems tend to converge (**Figure 3B**).

### 3.3 Metabolite profiling differences under different planting patterns

Metabolites extracted from the samples were analyzed using a high-resolution mass spectrometer in both positive and negative ion modes, generating comprehensive mass spectrometry datasets. The detailed statistical results of metabolite detection are presented in **Supplementary Table S2**. To ensure data quality, the total ion intensities of all detected metabolites in each sample were summed and quality validation was performed using total ion chromatograms (**Supplementary Figure S1**). To visualize the differences among sample groups, principal component analysis (PCA) and partial least squares-discriminant analysis (PLS-DA) were conducted on 16 samples in both positive and negative ion modes (**Supplementary Figure S2**). The PCA results showed a distinct separation between the two treatment groups along the first principal component (PC1), which accounted for 44.34% of the total variance, while PC2 explained an additional 20.19% of the variation. These results support the consistency and reliability of the analytical procedure. PLS-DA analysis further confirmed a clear separation between the intercropping and monoculture systems, highlighting the metabolic divergence induced by different planting patterns.

**Figure 4:**
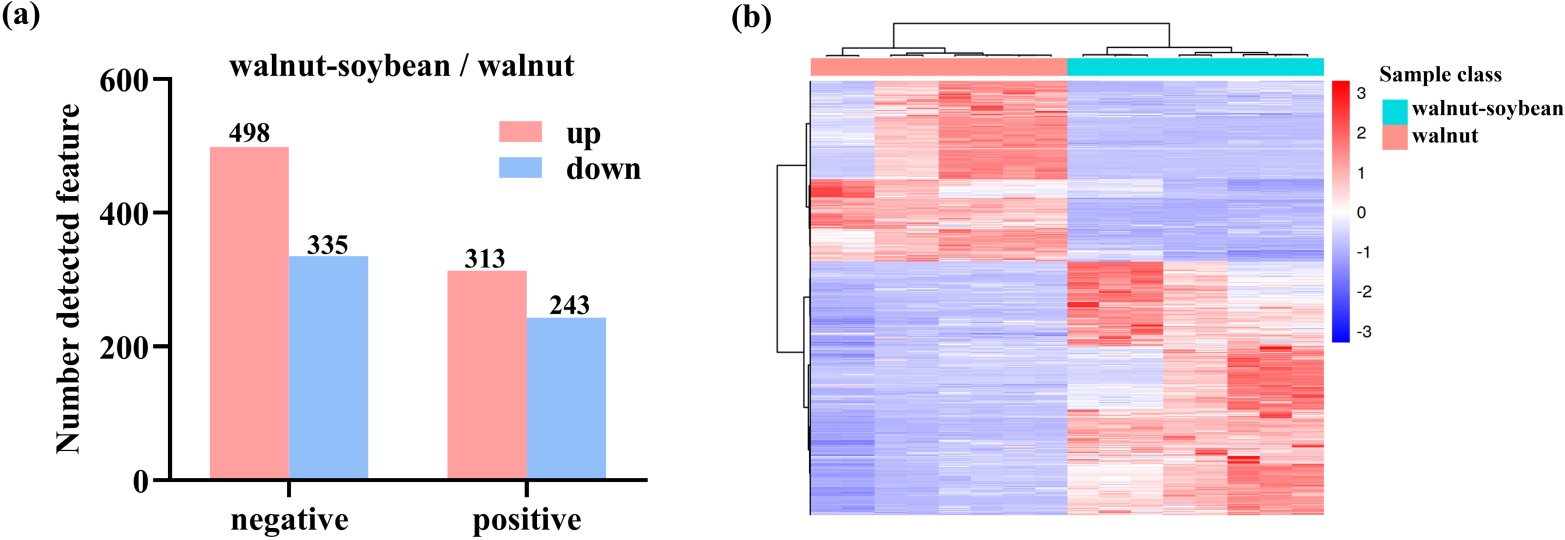
Metabolite profiles under different planting patterns. **(A)** Statistical chart of differential metabolites of walnut root systems under monoculture and intercropping conditions. **(B)** The heatmap shows the differential metabolites identified through statistical methods.

**Figure 4A** illustrates the number of differential metabolites identified in both positive and negative ion modes, clearly demonstrating significant metabolic differences between walnut intercropping and monoculture systems. Specifically, 833 and 556 metabolite ion features were closely associated with the two planting patterns under positive and negative ion modes, respectively. Notably, the number of upregulated metabolites was greater than that of downregulated ones under intercropping conditions compared to monoculture, suggesting that intercropping not only induces metabolic variation but also promotes the accumulation of specific metabolites. Furthermore, **Figure 4B** presents a hierarchical clustering heatmap, revealing a distinct separation of average metabolite profiles across the different experimental groups, thereby validating the observed metabolic distinctions.

### 3.4 Functional analysis of differential metabolites

**Figure 5:**
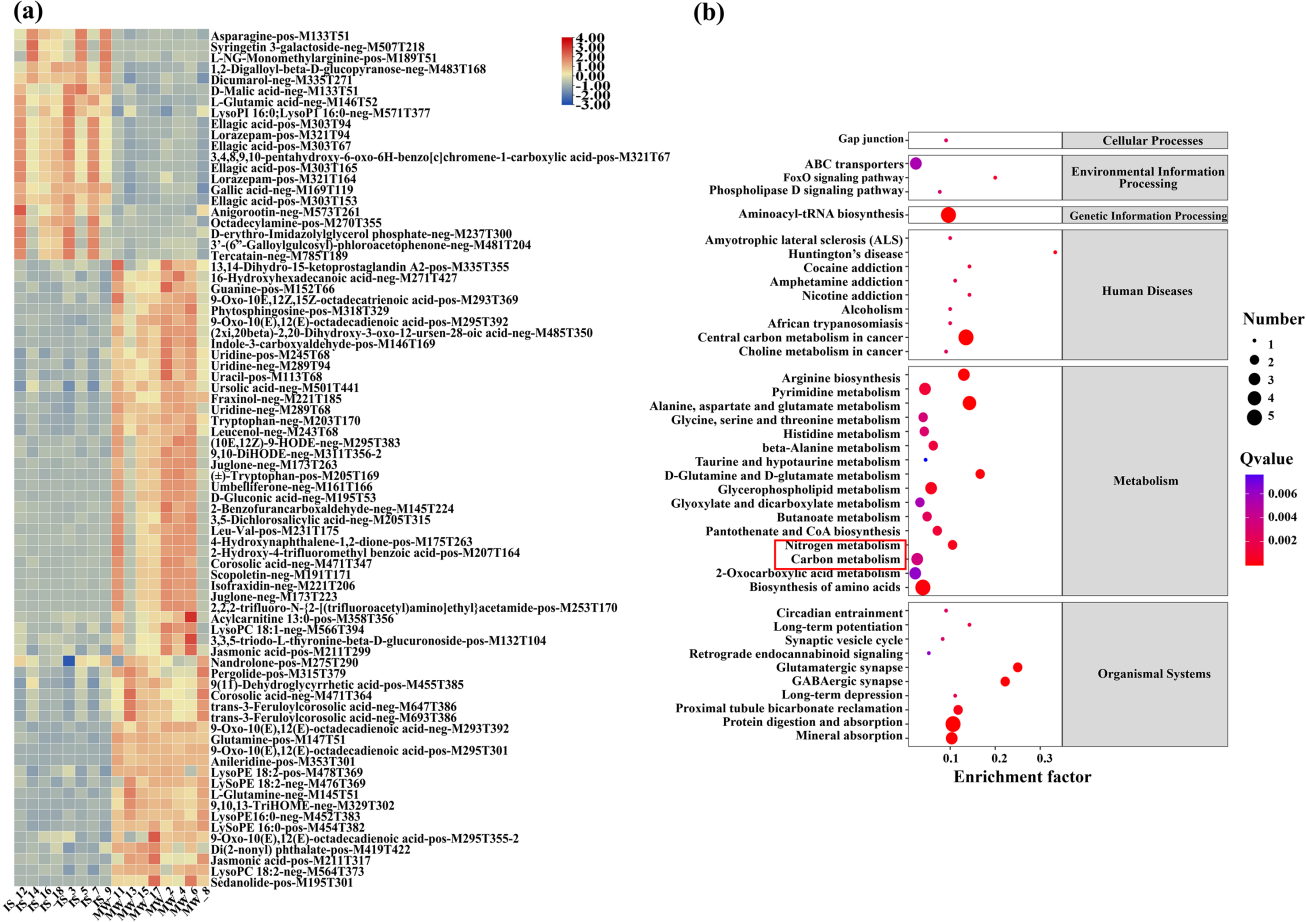
Analysis of differential metabolites in intercropping and monoculture systems. **(A)** The heatmap shows the differential metabolites with secondary identification. Each row represents a metabolite ion feature, and each column represents a sample. IS: Walnut-soybean intercropping system, MW: Walnut monoculture system. **(B)** Pathway enrichment analysis of secondarily identified metabolites annotated in the KEGG database. Results with a q-value < 0.05 are presented.

To examine the quantitative changes and identify differential metabolites, primary and secondary identification annotations were performed, and the secondary-identified differential metabolites were visualized (**Figure 5A**; **Supplementary Table S4**). The results demonstrated that intercropping with soybeans significantly altered the metabolite profiles in the walnut root system. A total of 80 differential metabolites were identified, primarily including lipids, nucleotides, phenylpropanoids, organic heterocycles, organic acids, and phenyl derivatives. Notably, the upregulated metabolites (22 in total) were predominantly phenylpropanoids, organic nitrogen compounds, organic acids, and lipids, while the downregulated metabolites (58 in total) were predominantly nucleotides, phenyl derivatives, and most lipids were significantly downregulated.

Subsequently, based on pathway classifications from the KEGG database, we conducted enrichment analysis on the secondary-identified differential metabolites (**Figure 5B**). All enriched metabolites were categorized into six major classes: metabolism, genetic information processing, environmental information processing, cellular processes, human diseases, and organismal systems. Among these, metabolic pathways exhibited the highest enrichment degree. The top seven enriched metabolic pathways included global and overview maps, lipid metabolism, amino acid metabolism, carbohydrate metabolism, other amino acid metabolism, nucleotide metabolism, and cofactor and vitamin metabolism. These pathways play fundamental roles in regulating cell proliferation, differentiation, and overall plant growth during walnut root development. Additionally, the pathway related to the biosynthesis of secondary metabolites and energy metabolism—ranked among the top enriched pathways, which is closely associated with the allelopathic effects of secondary metabolites, such as paeonol, secreted by walnut roots. KEGG enrichment analysis revealed that under intercropping conditions, pathways related to carbon and nitrogen metabolism, including amino acid metabolism, carbohydrate metabolism, and nucleotide metabolism, were significantly enriched. Collectively, these findings indicate that intercropping induces substantial alterations in carbon and nitrogen-related metabolic pathways within the walnut root system.

### 3.5 Transcriptome analysis of walnut roots under intercropping and monoculture conditions

A total of 259.46 million raw reads were generated through sequencing in this study. After rigorous quality filtering, the Q20 values (GC content-related) all surpassed 99.69%, while the Q30 values exceeded 92.03%, and the clean read ratio was above 96.47% (**Supplementary Table S3**). These metrics meet the stringent quality control standards for sequencing data. The Pearson correlation coefficients among the three biological replicates were high (**Supplementary Figure S3**), indicating robust data consistency and reliability, which to supports further analysis. The percentage of reads successfully mapped to the reference genome ranged from 87.13% to 93.15%, with the proportion of uniquely mapped reads falling between 58.89% and 62.98% (**Supplementary Table S5**).

**Figure 6:**
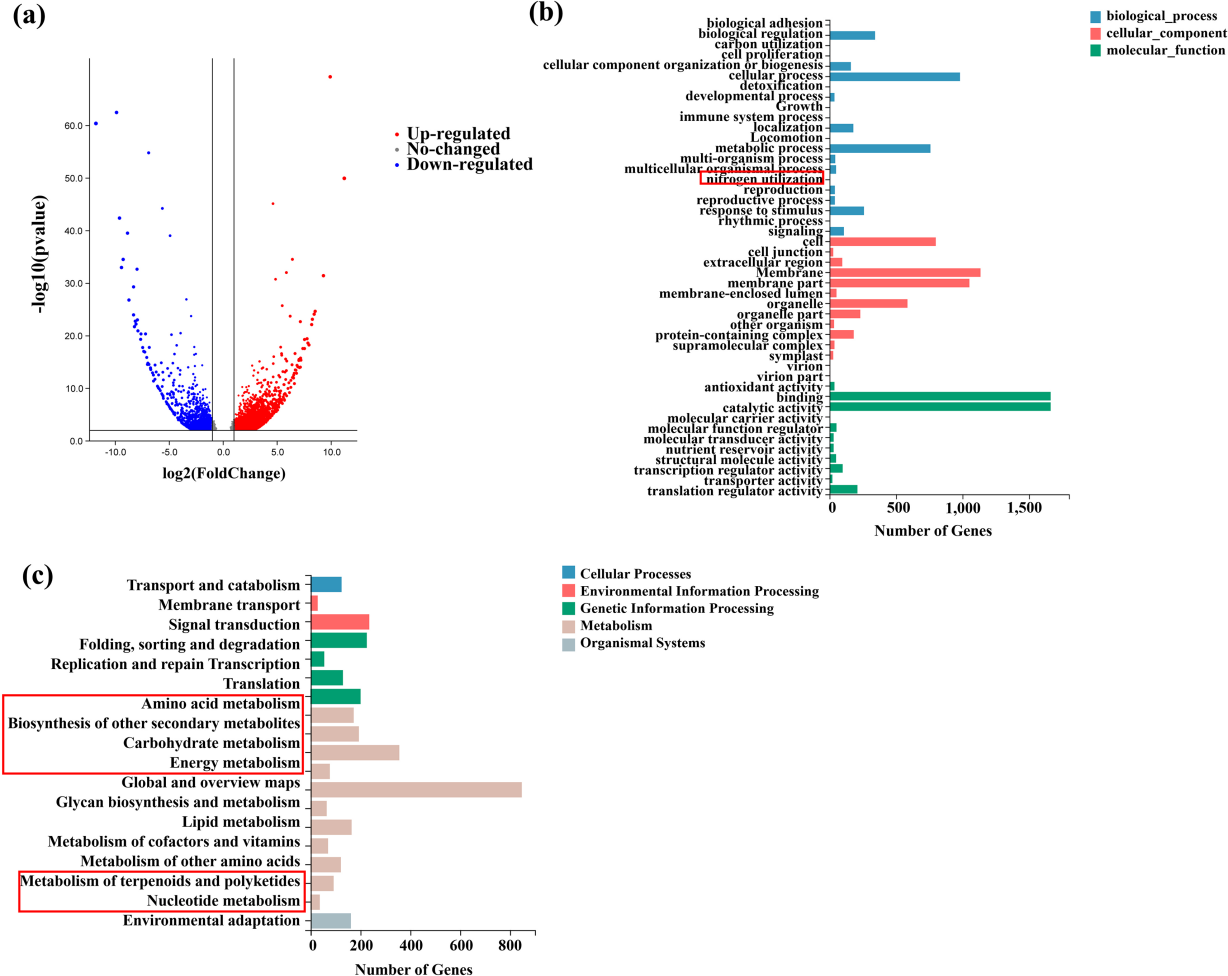
Transcriptome analysis of the walnut root system under intercropping. **(A)** Number of upregulated and downregulated differentially expressed genes (DEGs) in the walnut root system under intercropping. **(B)** The classification results of GO annotations for the differential genes. **(C)** The KEGG enrichment results of the differentially expressed genes in the walnut root system under intercropping. In the figure, the abscissa represents the number of DEGs annotated to each KEGG pathway, and the ordinate represents the KEGG pathway.

When comparing DEGs between walnut roots in the intercropping and monoculture systems, a total of 3,978 DEGs were identified, including 2,452 upregulated and 1,526 downregulated genes (**Figure 6A**). To elucidate the functional implications of these DEGs, Gene Ontology (GO) enrichment analysis was conducted on walnut roots under both planting conditions. Through this analysis, the DEGs were categorized into three main classes: biological processes, molecular functions, and cellular components. Notably, the term “nitrogen utilization” was significantly enriched, which is consistent with the findings from metabolomics analysis (**Figure 6B**).

The KEGG integrates genomic, chemical, and system-level functional information comprehensively. In this study, KEGG pathway enrichment analysis was performed to explore metabolic pathways in walnut root systems under different planting patterns. Following classification by pathway type, metabolic pathways exhibited the highest degree of enrichment. Consistent with the results of metabolomics analysis, several key pathways were significantly enriched, including global and overview maps, lipid metabolism, carbohydrate metabolism, amino acid metabolism, terpenoid and polyketide metabolism, as well as biosynthesis of other secondary metabolites. Additionally, pathways related to carbon and nitrogen metabolism, such as amino acid metabolism, sugar metabolism, and nucleic acid metabolism, were also significantly enriched (**Figure 6C**). Based on the integrated metabolomics and transcriptomics results, it can be concluded that walnut root systems under intercropping conditions undergo substantial alterations in carbon and nitrogen-related metabolic pathways.

### 3.6 Joint analysis of transcriptome and metabolome

**Figure 7:**
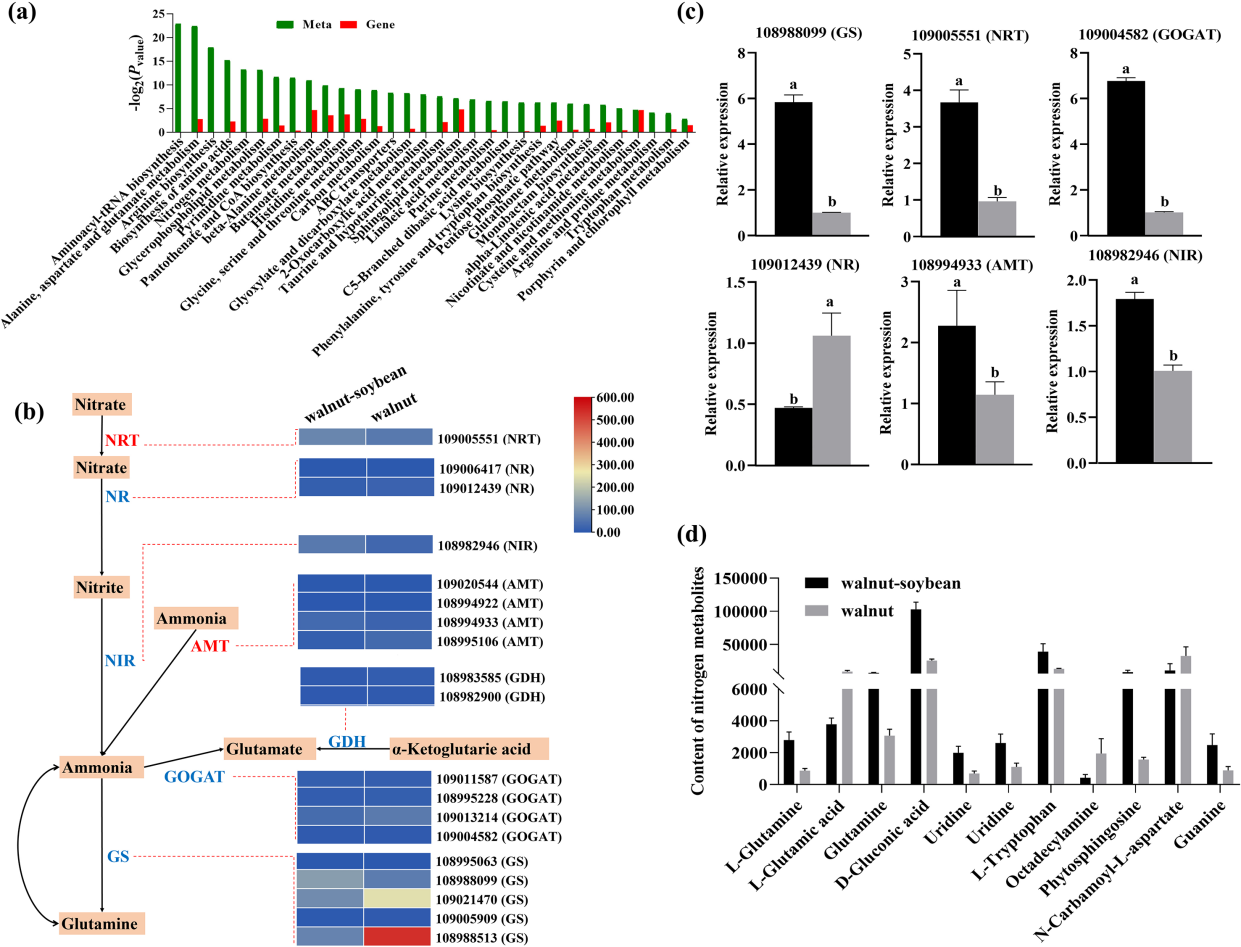
**(A)** Histogram of the combined KEGG enrichment p-values of differential metabolites and DEGs in the root system of walnuts. **(B)** Based on the RNA-seq results, the expression levels of nitrogen metabolism-related genes are shown through the heatmap of FPKM. The small squares in the figure represent the relative expression of genes in walnut-soybean intercropping and walnut monoculture systems. The red word represents the transporters. **(C)** qRT-PCR analysis revealed the expression levels of six key DEGs involved in the nitrogen metabolism pathway in the roots of walnut plants under intercropping and monoculture conditions. All data in the figure are presented as mean ± SD. Lowercase letters represent the significant difference within the same group (*p* < 0.05). **(D)** The changes in metabolites during the nitrogen metabolism pathway were summarized. The data were presented in the form of peak areas.

DEGs and differential metabolites were separately mapped to the KEGG pathway database to identify commonly enriched pathways shared between the two datasets, thereby elucidating the key biochemical and signal transduction pathways potentially involved in their regulatory mechanisms. A total of 32 co-enriched KEGG pathways were detected in both intercropping and monoculture systems (**Figure 7A**), with nitrogen metabolism being significantly enriched. Moreover, several pathways closely associated with carbon and nitrogen metabolism, including amino acid metabolism, sugar metabolism, and nucleic acid metabolism, also showed significant enrichment.

**Supplementary Table S6** provides a comprehensive list of differentially expressed genes related to nitrogen metabolism identified through GO enrichment analysis. **Figure 7B** illustrates the nitrogen metabolism pathway along with the heatmap representing the expression patterns of some genes. Key enzymes and transporters, including *NR*, *NIR*, *GOGAT*, *GDH*, *GS*, *nitrate transporter* (*NRT*), and *AMT* play essential roles in nitrogen metabolism. The expression trends of these genes in the intercropped walnut root system varied, reflecting their distinct functions and contributions to the nitrogen metabolic pathway. To validate the RNA-seq gene expression data presented in the heatmap, we conducted qRT-PCR for further confirmation. The results demonstrated that the expression levels of several critical genes in the walnut root system were significantly altered under intercropping compared to monoculture. Specifically, genes such as 108988099 (*GS*), 109005551 (*NRT*), 109004582 (*GOGAT*), 108994933 (*AMT*), and 108982946 (*NIR*) exhibited upregulation ranging from approximately 1.77-6.65 fold. Notably, the expression level of the 109012439 (*NR*) gene decreased after intercropping (**Figure 7C**). This downregulation may be attributed to feedback inhibition caused by increased NR enzyme activity in the walnut-soybean intercropping system. Consistently, the contents of several intermediate products involved in the nitrogen metabolism pathway, such as L-glutamine, glutamine, D-gluconic acid, uridine, L-tryptophan, phytosphingosine, and guanine, were markedly elevated in the walnut root intercropping system compared to the monoculture (**Figure 7D**). These findings suggest that nitrogen metabolism intermediates may play a crucial role in the walnut intercropping system.

### 3.7 The influence of intercropping on the enzymatic activities of walnut root systems

**Figure 8:**
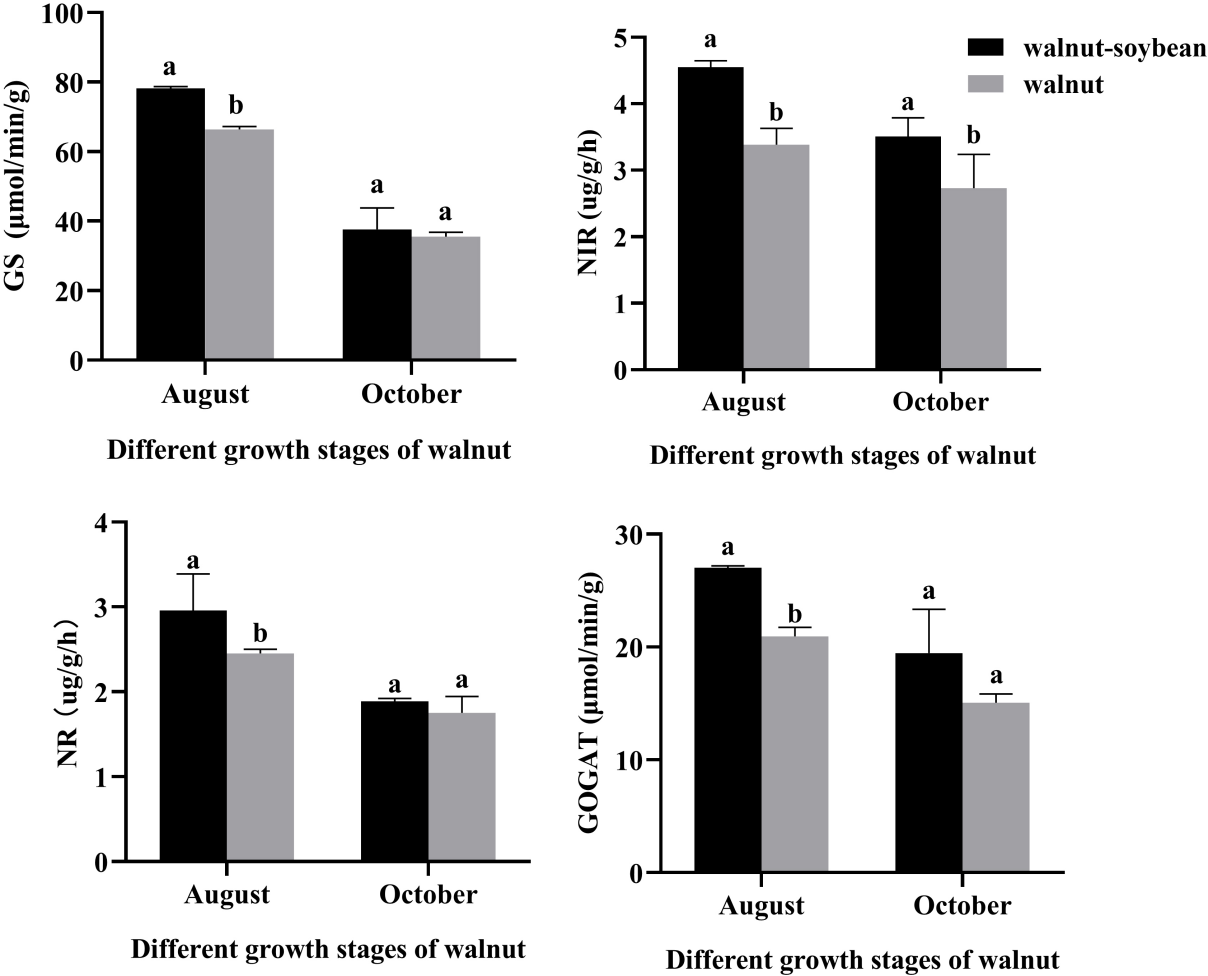
Detection of nitrogen metabolism enzyme activities (NR, NIR, GS, and GOGAT) in walnut root systems under monoculture and intercropping patterns in August and October. All data in the figure are indicated by mean ± SD. The small letters represent the significant difference at the same time point (*p* < 0.05).

As illustrated in **Figure 8**, under intercropping conditions, the enzymatic activities associated with nitrogen metabolism in walnut root systems exhibited varying degrees of change. The activities of the four analyzed enzymes were consistently higher in the intercropping treatment compared to the monoculture treatment. Specifically, significant differences in the activities of NR, GS, and GOGAT were observed between the intercropping and monoculture treatments in August, whereas no significant differences were noted in October. In contrast, NIR demonstrated significant differences between the two treatments even in October. These findings suggest that intercropping with soybeans enhances the GS/GOGAT cycle pathway, which is responsible for converting inorganic nitrogen to organic nitrogen in walnut root systems. Furthermore, these results consistent with the changes in nitrogen-related metabolic pathways identified through metabolomics analysis.

## 4 Discussion

Despite these insights, this study has inherent limitations due to the use of a planting bag system. First, the confined root space may restrict the root growth of walnuts and soybeans, altering interspecific interactions (e.g., competition) compared to field conditions and potentially exaggerating nutrient-related differences between intercropping and monoculture. Second, the planting bag environment lacks the heterogeneity, microbial complexity, and hydrological dynamics of field soil, disrupting stable nutrient feedbacks (e.g., nitrogen fixation, root exudation) and limiting the extrapolation of metabolic and gene expression results. Third, controlled conditions (uniform fertilization/irrigation, no abiotic stress) fail to simulate field variability (e.g., rainfall, temperature, soil nutrient heterogeneity). Pot-field differences are known to alter crop growth and nutrient use efficiency (Passioura, 2006). Thus, our findings represent an initial mechanistic exploration of intercropping-induced nutrient and metabolic dynamics. Future studies should validate these results under field conditions, with long-term monitoring of soil-plant-microbe interactions and environmental variables to enhance generalizability and practical relevance.

In intercropping systems, the root systems of trees and crops exhibit pronounced niche differentiation in spatial distribution. Specifically, tree roots, due to their longer lifespan, tend to penetrate deeper into the soil, whereas crop roots generally adopt a shallow distribution strategy to minimize spatial competition with tree roots (Jiang et al., 2018). This study further demonstrates that the vertical roots of intercropped walnut trees gradually shift downward during growth and are more developed compared to their horizontal roots. In contrast, the root systems of monocropped walnut trees exhibit a uniform distribution. An analysis of dry matter mass reveals that the intercropping pattern significantly enhances dry matter accumulation in walnut trees. Although leguminous plants are well-known for providing nitrogen through biological nitrogen fixation in crop-legume intercropping systems (Palmero et al., 2022), significant research gaps remain regarding nitrogen allocation in tree-legume intercropping systems. This study found that intercropping with legumes at different growth stages had differential effects on the total nitrogen content of both the aboveground parts and roots of walnut trees, thereby validating the feasibility of the tree-legume intercropping model. Intercropping orchards with legumes reduces the demand for nitrogen fertilizer through biological nitrogen fixation, improves nitrogen fertilizer use efficiency, and increases soil nutrient content (Inal et al., 2007; Li et al., 2011). Additionally, shifts in rhizosphere microbial assemblages under intercropping could promote plant nutrient uptake (Li et al., 2025). However, in grain-legume intercropping systems, the main enhancement of biodiversity and land sustainability has potential benefits for the generation of soil quality and fertility (Kaiqi et al., 2024). Analysis of soil nutrient dynamics showed higher soil organic matter content during the growth period of walnut trees, but similar levels between intercropping and monocropping during dormancy. Overall, intercropping patterns help improve soil nutrient conditions, laying a solid foundation for the healthy growth and high-quality, high-yield production of walnuts.

Metabolomics and RNA-seq technologies have emerged as pivotal omics tools for elucidating the mechanisms of environmental adaptation in biological systems (Parida et al., 2018; Feng et al., 2022). This study utilized LC-MS-based metabolomics and RNA-seq analyses to systematically investigate metabolic differences and transcriptomic profiles in walnut root systems under monoculture versus intercropping conditions, with the aim of uncovering their adaptive mechanisms to the intercropping environment. Metabolomics analysis identified 1,389 metabolite ion features that exhibited significant correlations with planting patterns. Transcriptomic profiling revealed 3,978 DEGs, including 2,452 upregulated and 1,526 downregulated genes. Prior research has demonstrated that waxy sorghum-soybean intercropping enhances nitrogen metabolism in waxy sorghum plants (Wang et al., 2025). In this study, GO and KEGG enrichment analyses highlighted the significant involvement of pathways related to carbon and nitrogen metabolism (e.g., amino acid, sugar, and nucleic acid metabolism) and nitrogen signal transduction in the intercropping system. Collectively, these findings reinforce the positive influence of intercropping on plant nitrogen metabolism.

Plant nitrogen metabolism is governed by key genes and enzymes involved in nitrate and ammonium transport and assimilation. Nitrate, the predominant form of nitrogen absorbed by plant roots, is primarily transported via NRTs (Sun et al., 2014), while ammonium transport relies predominantly on AMTs (Loqué and Von Wirén, 2004). Enzymes such as NR, NIR, GS, GOGAT, and GDH play critical roles in nitrogen metabolism (Balotf et al., 2016). Through transcriptomic analysis, this study explored the expression patterns of genes associated with nitrogen metabolism in walnut root systems under intercropping conditions. Results indicated differential expression of genes encoding *NR*, *NIR*, *GOGAT*, *GDH*, *GS*, *NRT*, and *AMT*, providing further evidence for the functional plasticity of the nitrogen metabolism pathway. qRT-PCR analysis further revealed that the expression levels of genes such as 108988099 (*GS*), 109005551 (*NRT*), 109004582 (*GOGAT*), 108994933 (*AMT*), and 108982946 (*NIR*) were significantly increased by approximately 1.77 to 6.65-fold. Notably, the expression level of the 109012439 (*NR*) gene was downregulated after intercropping. To explain this phenomenon, we propose several potential explanations: First, the nitrate absorption and utilization mechanisms in walnut roots within the intercropping system may differ from those observed in other cereal crops. Second, *NR* gene expression may be influenced by complex regulatory mechanisms, including post-transcriptional regulation, translational control, or interactions with other signaling pathways. Third, *NR* gene expression may be affected by the feedback inhibitory effect resulting from the enhanced enzyme activity of NR in the walnut and soybean intercropping system. Furthermore, intercropping may promote the accumulation of ammonium in the rhizosphere (achieved through the nitrogen fixation/microbial action of soybeans), thereby inhibiting the transcription of nitrate reductase. These hypotheses require further investigation and validation in future studies.

Extensive research has demonstrated that grain-legume intercropping systems enhance the activity of nitrogen metabolism-related enzymes. For instance, *Suryapani* et al. reported that wheat-lentil intercropping significantly increased NR, NIR, GS, and GOGAT activities in wheat leaves compared to monoculture wheat (Suryapani et al., 2013). Similarly, *Liu* et al. found that wheat-broad bean intercropping enhanced total GS and GOGAT activities in wheat flag leaves while upregulating the expression of *GS1*, *GS2*, *Fd-GOGAT*, and *NADH-GOGAT* genes (Liu et al., 2021). In this study, we observed for the first time that in a tree-legume intercropping system, walnut root NR, NIR, GS, and GOGAT activities were higher than those under monoculture conditions. This enhancement may be attributed to soybeans’ biological nitrogen fixation capacity, which increases nitrogen content in walnut tissues and improves nitrogen metabolism efficiency. Additionally, improvements in soil physicochemical properties and enzyme activities in the walnut-soybean intercropping system may have triggered the activation of walnut’s nitrogen metabolism system (Shao et al., 2023; Wang et al., 2025).

In summary, this study systematically analyzed the differences in root metabolites and transcriptomic profiles of walnuts grown under monoculture and intercropping systems, by integrating metabolomics and RNA-seq technologies. These findings not only provide robust evidence for understanding the physiological and biochemical responses in walnuts under different planting patterns, but also offer valuable insights for optimizing walnut cultivation practices to enhance yield and quality.

## 5 Conclusion

The walnut-soybean intercropping system significantly enhances the nitrogen metabolism in walnut roots. Intercropping not only promotes the vertical growth of walnut roots and improves dry matter accumulation but also modifies nitrogen distribution, resulting in increased nitrogen content in aboveground parts during dormancy and in the roots during the hard kernel stage and winter dormancy period. Additionally, the intercropping system increases soil organic matter content, thereby providing better nutrition for walnut growth. This study establishes the first comprehensive metabolomic and transcriptomic database for analyzing the metabolic and transcriptional profiles of walnut roots in the walnut-soybean intercropping system. Metabolomic analysis reveals that intercropping activates carbon-nitrogen metabolic pathways and nitrogen transmembrane transport pathways. Transcriptomic analysis successfully identifies approximately 3,978 DEGs. A total of 19 DEGs involved in nitrogen metabolism-related pathways were identified as candidate genes based on their expression pattern. Furthermore, enzyme activity validation demonstrates that intercropping significantly enhances the activities of key enzymes such as GS and GOGAT, thereby promoting the conversion of inorganic nitrogen into organic nitrogen.

## Data Availability

Raw data have been deposited to National Center for Biotechnology Information (NCBI) under the BioProject number PRJNA1404689.

## Supplementary material

Supplementary Figure 1 The total ion chromatograms of positive ions **(a)** and negative ions **(b)** detected by LC-MS in the root samples of walnut grown in intercropping and monoculture.

Supplementary Figure 2 Quantitative quality control of differential metabolites. **(a)** PCA score plots of each pair of phenotypes in each comparison group. **(b)** PLS-DA score plots of each pairwise phenotype in the comparison groups.

Supplementary Figure 3 Pearson correlation between samples for RNA-Seq. “walnut-soybean” represents the root system samples of intercropped soybeans with walnuts; “walnut” represents the root system samples of the monocropped walnuts.

Supplementary Table 1 The primers used for qRT-PCR.

Supplementary Table 2 Total ion number and identification statistics.

Supplementary Table 3 Quality statistics of filtered Reads.

Supplementary Table 4 The different substances with significant differences in secondary identification during metabolite analysis.

Supplementary Table 5 Summary of the sequencing data generated for RNA-Seq and mapping on the walnut genome.

Supplementary Table 6 Differentially expressed genes related to nitrogen metabolism.
