# Supplementary figures and images for: Integrated transcriptomic and metabolic profiling reveals the molecular mechanism of improved nitrogen metabolism in walnut (Juglans regia L.) roots mediated by soybean intercropping
Source: Front Plant Sci. 2026 Jan 29;16:1658364. doi: 10.3389/fpls.2025.1658364 (PMC12894250; doi:10.3389/fpls.2025.1658364)

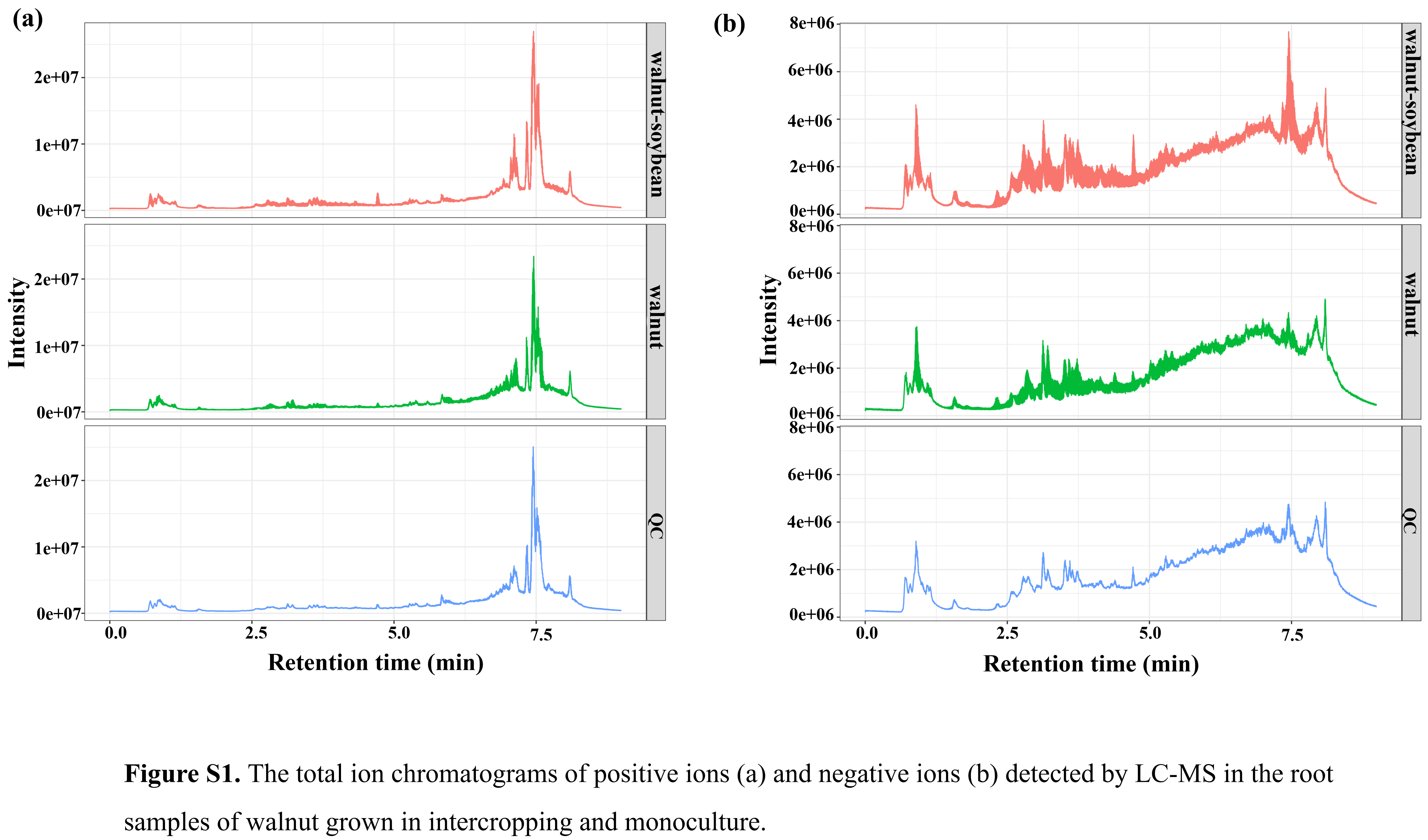

Supplement: Supplementary Figure 1 — The total ion chromatograms of positive ions (a) and negative ions (b) detected by LC-MS in the root samples of walnut grown in intercropping and monoculture. [file Image1.jpeg]

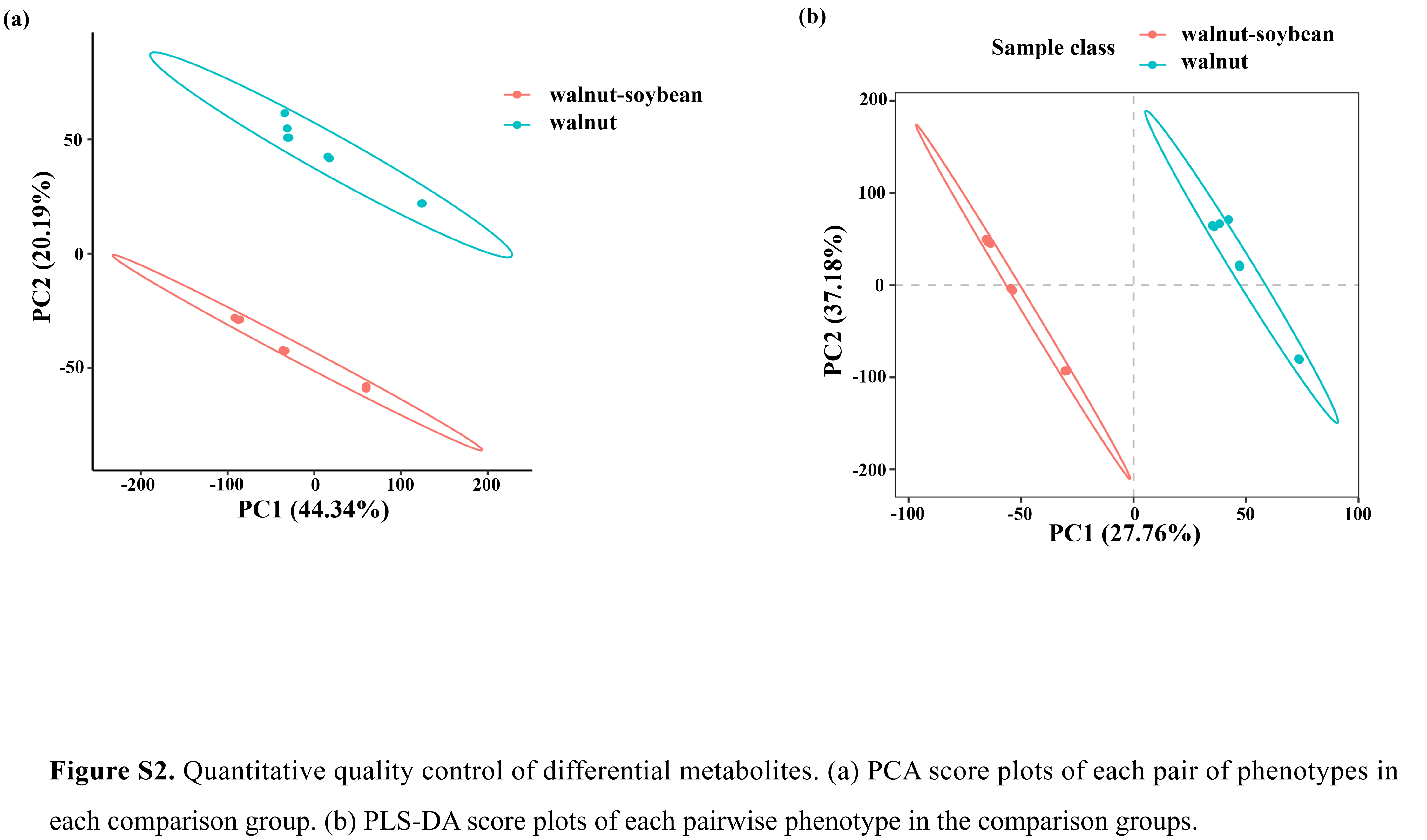

Supplement: Supplementary Figure 2 — Quantitative quality control of differential metabolites. (a) PCA score plots of each pair of phenotypes in each comparison group. (b) PLS-DA score plots of each pairwise phenotype in the comparison groups. [file Image2.jpeg]

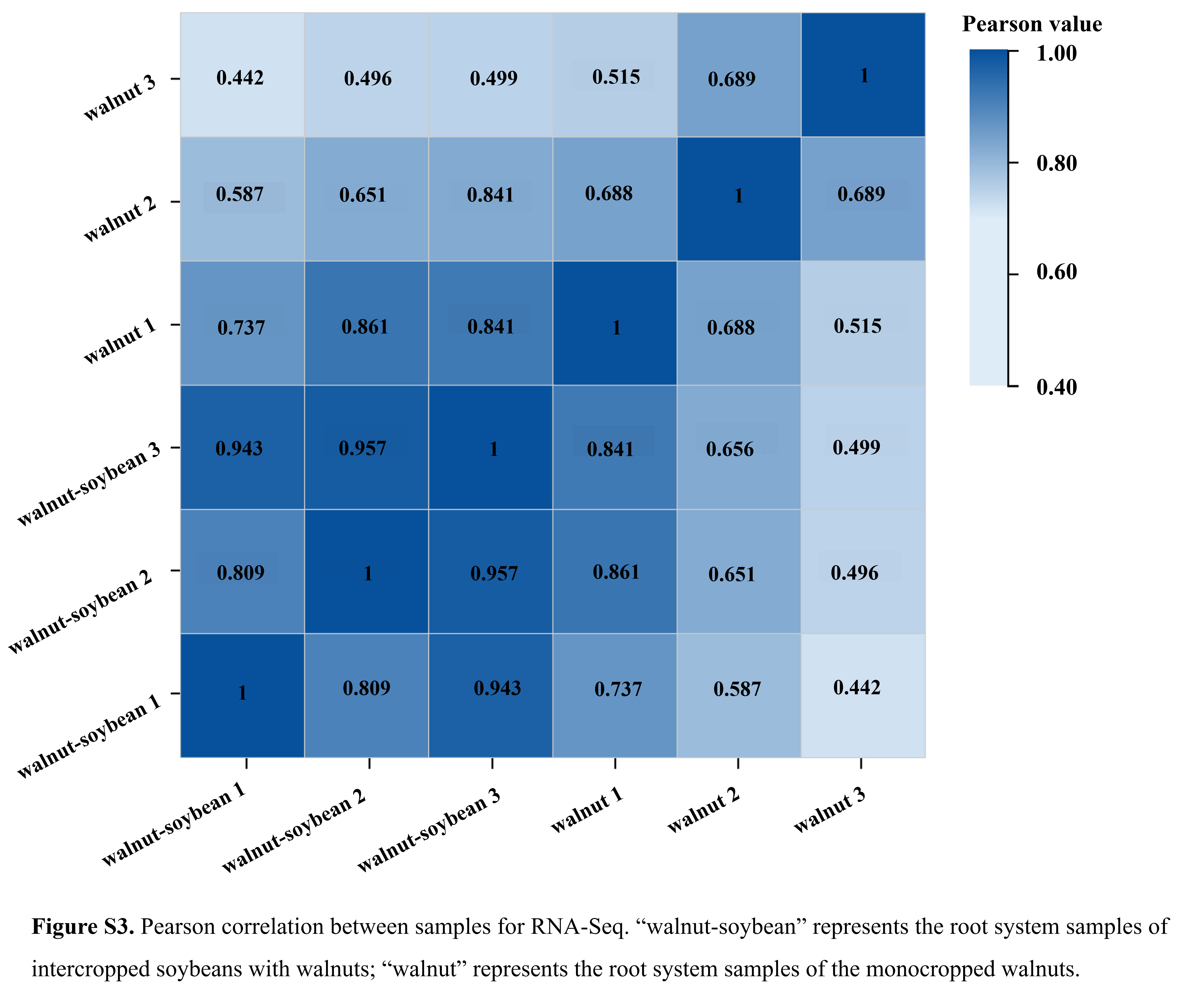

Supplement: Supplementary Figure 3 — Pearson correlation between samples for RNA-Seq. “walnut-soybean” represents the root system samples of intercropped soybeans with walnuts; “walnut” represents the root system samples of the monocropped walnuts. [file Image3.jpeg]
